# Health Inequalities among Workers with a Foreign Background in Sweden: Do Working Conditions Matter?

**DOI:** 10.3390/ijerph10072871

**Published:** 2013-07-10

**Authors:** Andrea C. Dunlavy, Mikael Rostila

**Affiliations:** Centre for Health Equity Studies (CHESS), Stockholm University/Karolinska Institute, SE-106 91 Stockholm, Sweden; E-Mail: mikael.rostila@chess.su.se

**Keywords:** health inequalities, working conditions, foreign background populations, social determinants of health, Sweden

## Abstract

Employment and working conditions are key social determinants of health, yet current information is lacking regarding relationships between foreign background status, working conditions and health among workers in Sweden. This study utilized cross-sectional data from the 2010 Swedish Level of Living Survey (LNU) and the Level of Living Survey for Foreign Born Persons and their Children (LNU-UFB) to assess whether or not health inequalities exist between native Swedish and foreign background workers and if exposure to adverse psychosocial and physical working conditions contributes to the risk for poor health among foreign background workers. A sub-sample of 4,021 employed individuals aged 18–65 was analyzed using logistic regression. Eastern European, Latin American and Other Non-Western workers had an increased risk of both poor self-rated health and mental distress compared to native Swedish workers. Exposure to adverse working conditions only minimally influenced the risk of poor health. Further research should examine workers who are less integrated or who have less secure labor market attachments and also investigate how additional working conditions may influence associations between health and foreign background status.

## 1. Introduction

Recent studies have suggested that socioeconomic health inequalities are increasing across Europe and in the United States [1,2]. Even in modern welfare states such as Sweden, in which governmental policies have been shown to reduce inequalities on a number of social and economic outcomes [3], health disparities persist. There are multiple mechanisms through which such inequalities can arise, including differences in early life experiences, access to health care, living conditions and psychosocial environments, as well as differences in education, occupation, and income [4,5]. Yet one of the foremost factors among these social determinants of health is employment and working conditions [6]. While stable employment and good working conditions can provide individuals with essential health promoting assets such as financial security, opportunities for personal development and self-esteem [7], exposure to adverse working conditions can negatively impact both physical [8,9,10,11] and mental health [9,12] and may serve to create or exacerbate health inequalities.

Several studies have suggested that health inequalities in Sweden may also exist by migration background status, with foreign born persons often experiencing poorer health outcomes and higher mortality rates than the native born population [13,14,15,16,17,18]. However, results have been mixed, with some studies finding lower mortality rates [19] and a lower risk of illness [19,20] among certain foreign born populations when compared to native born Swedes. Foreign born persons and their children currently comprise 19.1% of the total Swedish population [21], and the number of working age foreign born persons migrating to Sweden is expected to increase [22]. Yet the roles that migration and ethnicity play in the formation and maintenance of health inequalities have often been overshadowed in European health equity research by socioeconomic determinants of health [23], and limited knowledge exists regarding migration related factors that may contribute to the health differentials found among foreign background groups.

Foreign born workers in Sweden have also been shown to have poorer working conditions than those who are native born [24,25,26] and are more likely to suffer from a marginalized status in the labor market [24,25,26,27,28]. As such there is reason to believe that exposure to adverse working conditions may partially explain existing health inequalities between native and foreign born workers in Sweden. Yet despite evidence which demonstrates the importance of both labor market participation and working conditions to migrant integration, health and well-being [29,30,31], only a paucity of research has empirically examined the contribution of working conditions to health outcomes among foreign background groups in Sweden.

Given the projected growth of foreign born populations in Sweden and disparities in both health outcomes and exposure to adverse working conditions between foreign and native born groups, relationships between migration, health, and the work environment require further examination. The aims of the present study are therefore to: (1) describe the distribution of adverse psychosocial and physical working conditions among native and foreign background workers in Sweden; (2) analyze the risk for poor health outcomes among foreign background workers compared to that of native workers; and (3) determine if exposure to adverse working conditions may influence associations between health and foreign background status.

## 2. Methods

### 2.1. Survey Data

Ethical approval for this research was granted by The Swedish Research Council (Vetenskapsrådet, ethical approval number 2012/1260-31). Data were derived from the 2010 wave of the Swedish Level of Living Survey (LNU) and the Level of Living Survey for Foreign Born Persons and their Children (LNU-UFB; LNU-UFB data have not been officially released at the time of this writing. The authors take responsibility for the quality of the data presented here, and do not expect conclusions to change when the data is officially released). The LNU data is a representative sample of the entire Swedish population aged 18–75. The 2010 wave of the LNU represents the sixth iteration of this survey since its original inception in 1968. The survey has a panel design so that longitudinal comparisons can be made, however the 2010 iteration also includes a new selection of younger participants (aged 18–28 years) and persons who have immigrated to Sweden since 2000 so that it remains cross-sectionally representative. Face to face and telephone interviews were conducted in 2010–2011 and administered by trained interviewers from Statistics Sweden (Statistiska centralbyrån, SCB). The non-response rate for the LNU 2010 survey was 38.5%, and in total 5,344 interviews were conducted.

The LNU-UFB utilized Swedish registry data to select a representative sample of the entire foreign born population aged 18–75 who had lived in Sweden for at least five years. From this representative selection a stratified sampling technique was then utilized in order to ensure that a statistically sufficient number of foreign born persons from different regions of origin were represented in the data. The sampling frame included seven region of origin groups, each of which were divided into three age categories (18–30 years, 31–55 years, and 56–74 years). Each age category was comprised of 350 potential respondents, and each region of origin group was composed of 1,050 persons who were approached to participate. In total, 3,451 face to face interviews were conducted between 2010–2012 by trained interviewers from SCB. The non-response rate was 50.0%. In the vast majority of cases interviews were conducted in Swedish, however 5% of the interviews were conducted in a different language; 3.5% of these interviews were conducted with the aid of an interpreter, and the remaining 1.5% were administered with the aid of a close relative of the respondent who assisted with translation. The LNU-UFB is a new survey dataset which was designed to exclusively examine the living conditions of foreign born persons in Sweden and contains questions which are identical to those found in the LNU 2010 data, as well as additional items related to migration experiences, language abilities, employment and social networks in the country of origin and in Sweden.

### 2.2. Study Population

Data on native born workers from the LNU 2010 was combined with data on foreign born workers from the LNU-UFB. LNU 2010 respondents who were foreign born were excluded from the current study. Unemployed individuals were also excluded, as were the self-employed and farmers, as differences in the type and severity of work related stressors may differ between those who are self-employed or farmers and those in more traditional workplace environments. The total study population (n = 4,021) was comprised of currently employed adults aged 18–65, working at least 20 hours per week. The present study utilized 66% of the LNU 2010 interviews and 51% of the LNU-UFB interviews as described above.

### 2.3. Health Outcomes

The health outcomes evaluated in this study were self-rated health and mental distress. Self-rated health has previously been correlated with morbidity and mortality outcomes [32,33] and as such may be considered a robust, valid indicator of overall health status. Self-rated health was assessed by the question, *‘How would you rate your own overall health?’*. Response items were *‘good’*, *‘bad’*, and *‘something in between’*. Individuals who rated their health as *‘bad’* or *‘something in between’* were considered to have poor self-rated health. Mental distress was measured with an additive index of select items from a checklist of various mental health symptoms experienced in the last 12 months, and included: *‘general tiredness’*, *‘insomnia’*, *‘nervous troubles (anxiety, uneasiness, anguish)’*, ‘*depression, deep dejection’*, and *‘overexertion’*. Response options were *‘no’*, *‘yes, mild’*, and *‘yes, severe’*, with ‘*no*’ responses assigned zero points, *‘yes, mild’* responses one point, and *‘yes, severe’* responses three points. Participants scoring three or more points on the index (*i.e.*, one severe or three mild mental health ailments) were coded as having mental distress. This mental distress scale has been used in previous research utilizing LNU survey data [12,34].

### 2.4. Migration Background

Respondents were divided into six categories according to region of origin. A single item on the LNU-UFB queried individuals about their country of birth and was used to categorize foreign born respondents into four groups: *Western*, which included persons from Western Europe and other Western OECD (Organization for Economic Cooperation and Development) member states; *Eastern European*, which comprised Eastern European and former Soviet countries; *Latin American*, which consisted of countries in Central and South America and the Caribbean; and *Other Non-Western*, which included countries in Asia, Northern Africa, the Middle East and Sub-Saharan Africa. Native background status was derived from LNU 2010 survey questions concerning the country and/or county respondents grew up in *(‘In what country/county did you live during your childhood, i.e., up to age 16?’*) and the nationality of their parents (*‘What nationality is/was your mother/father?’*). Persons born in Sweden with two native born parents were coded as *Native Swedish*. Persons born in Sweden with either two foreign born parents or one native born and one foreign born parent were coded as *Foreign Parentage Swedish.*

### 2.5. Working Conditions

Psychosocial working conditions were operationalized in accordance with the Demand Control Model (DCM) [35]. This model has been used extensively in studies of work-related health outcomes, and is comprised of two main components which the model maintains can impact work related stress and subsequent health outcomes: *psychosocial demands* and *decision latitude*. Psychosocial demands refer to the time pressures and mental demands experienced by workers; decision latitude refers to an individual’s control over how and when to complete work tasks and how skills are utilized on the job. Workplace social support was also assessed as an additional component of the DCM, as high support may have a protective effect against stressful working conditions [36,37]. Under the DCM the interaction of high demands and low decision latitude is considered to result in *high strain jobs*, which are seen as the most health-deleterious. However, some prior research has found no or partial interaction effects of these components [30,38] and as such we have assessed the model constructs separately in order to obtain a more detailed understanding of the independent effect of each.

The LNU and LNU-UFB surveys assess DCM constructs with four proxy measures of psychosocial demands and decision latitude in the workplace. The items *‘Is your work mentally demanding?’* and *‘Is your work hectic or stressful?’* assessed demands, and respondents answering *‘yes’* to both of these items were coded as having high psychosocial demands. The items *‘Can you decide your own work pace?’* and *‘Is your work monotonous?’* measured decision latitude. Respondents who reported being able to decide their own work pace and not having monotonous jobs were coded as having high decision latitude. Level of social support was assessed on a five point scale with the item, *‘To what extent does your work entail that you can get support and help from workmates if needed?’*. Responses were dichotomized and the responses ‘*to a small extent’* and *‘not at all’* were coded as low support, while ‘*to a very large extent’*, *‘to a large extent’*, and *‘to a certain extent’* were coded as high support. It should also be mentioned that the LNU survey was utilized in the initial development of the DCM [39].

Although not accounted for by the DCM, physical working conditions also represent an important aspect of working life. Exposure to physically demanding work has been shown to impact health, both directly [40] and indirectly via stress-mediated pathways [8]. Physical working conditions were measured with two dichotomous (yes/no) items which assessed if respondents’ work entailed: (1) ergonomic stress (‘*Does your job require you to adopt unsuitable/uncomfortable working postures?’*); or (2) physical demands (‘*Is your work physically demanding in any other way?’*).

### 2.6. Statistical Analyses

Data were analyzed using logistic regression models to estimate odds ratios (OR) for poor self-rated health and mental distress with Native Swedish workers used as the reference category. In order to account for differences in the sampling strategies used across the two datasets we utilized a weight variable in the analyses. Potential socio-demographic confounders including age, sex, occupational class and civil status at the time of interview were also assessed and included in regression models. These variables were selected for inclusion in the models as they have previously been shown to influence health outcomes [4,41]. Age was measured as a continuous variable and civil status as a dichotomous variable (single/cohabitating partner). Occupational class was assessed according to the Swedish socioeconomic classification system (SEI). Respondents were coded as belonging to one of the following five categories: unskilled workers, skilled workers, lower non-manuals, mid-level non-manuals, and higher non-manuals. Additional variables included in the models were: (1) number of weekly working hours, as amount of time at work can influence exposure to adverse working conditions, and (2) reason for migration, in terms of whether or not respondents had migrated to Sweden for purposes of work or studies (yes/no), as health status may differ among labor migrants or international students compared to those who migrate for other reasons (e.g., asylum seekers).

In total seven regression models were calculated. Model 1 was adjusted for age, sex, occupational class, civil status, weekly working hours and migration reason and examined whether or not health inequalities exist between groups. Models 2–6 controlled for the aforementioned potential confounders and also evaluated the independent associations of each working condition assessed with both poor self-rated health and mental distress. In Model 7 all working conditions were placed into the model to examine fully adjusted associations with poor health outcomes. Explained fractions (XF) were utilized to estimate the influence of adverse working conditions on excess risk for poor self-rated health and mental distress. Explained fractions were calculated by using the OR of risk for poor health for each country of origin group before adjustment for working conditions in Model 1 (ORa) and the OR of risk for poor health for each country of origin group after adjustment for working conditions (ORb), in both the independent (Models 2–6) and fully adjusted (Model 7) models. The equation utilized to calculate XF was:

[XF = (ORa-1) − (ORb − 1)/ (ORa − 1)]

## 3. Results

Table 1 shows proportions of the socio-demographic characteristics, adverse working conditions and poor health outcomes of workers by region of origin. Eastern Europeans reported the greatest proportion of poor self-rated health (30.6%) as well as mental distress (20.5%). Approximately 50% of workers in all categories experienced high psychosocial demands, although demands were experienced to a slightly lesser degree among the Other Non-Western group (45.5%). However, the Other Non-Western group reported the highest level of low decision latitude (59.7%) followed by the Foreign Parentage Swedish (53.9%) and Latin American (52.0%) groups. Latin American workers also reported the highest proportion of low workplace social support (31.7%). Approximately 30–40% of all workers reported ergonomic stress, with Foreign Parentage Swedish workers reporting the highest rates (41.2%). Workers from Other Non-Western countries reported the most physical demands (54.1%), followed by Foreign Parentage Swedish and Latin American workers (53.4% and 52.2% respectively). Chi-square tests revealed that significant differences in exposure to adverse working conditions existed between groups on all conditions except for low social support (χ^2^ = 0.41) and ergonomic stress (χ^2^ = 0.07), and significant differences between groups were also found for both poor health outcomes (data not shown).

Table 2 shows OR of poor self-rated health by region of origin. After adjustment for age, sex, occupational class, civil status, weekly working hours and migration reason (Model 1) Eastern European, Latin American and Other Non-Western employees were found to have increased odds of poor self-rated health when compared to Native Swedish workers. Associations remained significant in all subsequent models adjusted for working conditions (Models 2–7). All working conditions were found to be significantly associated with poor self-rated health in all models except Model 7, the fully adjusted model, in which low decision latitude and ergonomic stress were not significant and low social support only showed a trend towards significance (*p* = 0.059).

**Table 1:** Background variables and prevalence of adverse working conditions and poor health outcomes by region of origin *.

	Native Swedish	Foreign Parentage Swedish	Western	Eastern European	Latin American	Other Non-Western	Total
**Total, n (%)**	2,055 (83.2)	216 (8.7)	582 (2.7)	264 (2.2)	294 (0.6)	610 (2.5)	4,021 (100.0)
**Gender, n (%)**							
Men	1,077 (52.3)	105 (49.3)	266 (42.4)	119 (44.3)	131 (46.1)	295 (48.9)	1,993 (51.5)
Women	978 (47.7)	111 (50.7)	316 (57.6)	145 (55.7)	163 (53.9)	315 (51.1)	2,028 (48.5)
**Age, m (SD)**	43.7 (12.1)	36.2 (11.5)	50.3 (9.3)	435 (11.3)	44.9 (10.9)	42.2 (10.3)	43.1 (12.2)
**Average Weekly Working Hours, m (SD)**	38.0 (5.5)	38.0 (6.1)	38.0 (5.8)	38.5 (6.1)	38.3 (6.0)	37.8 (5.3)	38.0 (5.6)
**Civil Status, n (%)**							
Single	540 (26.2)	90 (42.5)	165 (24.6)	80 (26.0)	98 (29.2)	225 (30.4)	1,198 (27.7)
Cohabitating Partner	1,515 (73.9)	126 (57.5)	417 (75.5)	184 (74.0)	196 (70.8)	385 (69.7)	2,823 (72.3)
**Occupational Class, n (%)**							
Unskilled workers	356 (17.4)	66 (29.9)	108 (18.0)	82 (30.7)	88 (28.0)	238 (38.3)	938 (19.4)
Skilled workers	351 (17.1)	31 (14.0)	101 (21.2)	53 (20.8)	71 (20.6)	143 (22.4)	750 (17.2)
Lower non-manuals	320 (15.6)	38 (17.2)	65 (9.8)	26 (8.8)	26 (9.0)	57 (8.3)	532 (15.2)
Mid-level non-manuals	577 (28.2)	58 (26.2)	156 (26.9)	59 (22.6)	74 (27.7)	103 (19.3)	1,027 (27.6)
Higher non-manuals	446 (21.8)	28 (12.7)	152 (24.2)	44 (17.1)	35 (14.7)	69 (11.7)	774 (20.6)
**Poor Health Outcomes, n (%)**							
Poor self-rated health	339 (16.4)	44 (20.8)	97 (20.1)	75 (30.6)	64 (23.7)	152 (26.7)	771 (17.5)
Mental distress	239 (11.5)	30 (14.9)	78 (11.9)	55 (20.5)	57 (18.7)	94 (16.4)	553 (12.2)
**Adverse Working Conditions, n (%)**							
High psychosocial demands	1,048 (51.1)	105 (48.4)	282 (47.7)	137 (52.9)	153 (53.0)	264 (45.5)	1,989 (50.7)
Low decision latitude	925 (44.9)	113 (53.9)	254 (42.9)	118 (45.0)	154 (52.0)	365 (59.7)	1,929 (46.1)
Low social support	517 (25.3)	57 (26.2)	151 (26.3)	59 (21.9)	87 (31.7)	148 (27.1)	1,019 (25.4)
Ergonomic stress	739 (35.9)	88 (41.2)	176 (35.8)	98 (37.8)	97 (32.8)	209 (37.1)	1,407 (36.4)
Physically demanding work	824 (40.0)	113 (53.4)	236 (42.0)	117 (46.5)	166 (52.2)	337 (54.1)	1,793 (41.8)

* weighted percentages, means and standard deviations provided in order to account for differences in sampling techniques utilized by the LNU 2010 and LNU-UFB surveys.

**Table 2:** Multivariate regression analysis of risk for poor self-rated health among foreign background workers in Sweden.*

	Model 1		Model 2		Model 3		Model 4		Model 5		Model 6		Model 7	
	OR (95% CI)	Pv	OR (95% CI)	Pv	OR (95% CI)	Pv	OR (95% CI)	Pv	OR (95% CI)	Pv	OR (95% CI)	Pv	OR (95% CI)	Pv
**Country of origin**														
Native Swedish	1.00		1.00		1.00		1.00		1.00		1.00		1.00	
Foreign Parentage Swedish	1.42 (0.99–2.05)	0.059	1.44 (1.00–2.08)	0.052	1.41 (0.98–2.03)	0.067	1.41 (0.98–2.04)	0.064	1.42 (0.99–2.05)	0.058	1.39 (0.96–2.00)	0.082	1.40 (0.96–2.03)	0.076
Western	1.27 (0.91–1.78)	0.161	1.28 (0.92–1.80)	0.148	1.27 (0.90–1.78)	0.171	1.28 (0.91–1.79)	0.156	1.27 (0.91–1.78)	0.161	1.25 (0.89–1.75)	0.190	1.27 (0.90–1.79)	0.172
Eastern European	2.39 (1.74–3.28)	0.000	2.37 (1.72–3.26)	0.000	2.42 (1.77–3.32)	0.000	2.41 (1.76–3.31)	0.000	2.44 (1.78–3.34)	0.000	2.42 (1.76–3.33)	0.000	2.45 (1.78–3.37)	0.000
Latin American	1.50 (1.06–2.12)	0.022	1.48 (1.05–2.09)	0.027	1.48 (1.05–2.10)	0.025	1.47 (1.04–2.08)	0.030	1.55 (1.09–2.19)	0.014	1.47 (1.03–2.08)	0.031	1.44 (1.01–2.06)	0.043
Other Non-Western	1.79 (1.34–2.40)	0.000	1.80 (1.35–2.42)	0.000	1.76 (1.31–2.35)	0.000	1.78 (1.32–2.38)	0.000	1.86 (1.38–2.49)	0.000	1.79 (1.34–2.41)	0.000	1.79 (1.33–2.42)	0.000
**High Psychosoc. Demands**			1.48 (1.20–1.82)	0.000									1.37 (1.11–1.69)	0.003
**Low Decision Latitude**					1.30 (1.05–1.60)	0.016							1.16 (0.93–1.43)	0.186
**Low Social Support**							1.27 (1.01–1.60)	0.041					1.25 (0.99–1.58)	0.059
**Ergonomic Stress**									1.35 (1.07–1.69)	0.011			1.13 (0.87–1.46)	0.363
**Physical Demands**											1.54 (1.22–1.94)	0.000	1.37 (1.06–1.79)	0.018

* model adjusted for age, sex, occupational class, civil status, weekly working hours and migration reason.

**Table 3:** Multivariate regression analysis of risk for mental distress among foreign background workers in Sweden *.

	Model 1		Model 2		Model 3		Model 4		Model 5		Model 6		Model 7	
	OR (95% CI)	Pv	OR (95% CI)	Pv	OR (95% CI)	Pv	OR (95% CI)	Pv	OR (95% CI)	Pv	OR (95% CI)	Pv	OR (95% CI)	Pv
**Country of origin**														
Native Swedish	1.00		1.00		1.00		1.00		1.00		1.00		1.00	
Foreign Parentage Swedish	1.15 (0.77–1.73)	0.489	1.18 (0.78–1.79)	0.432	1.14 (0.76–1.72)	0.522	1.14 (0.76–1.72)	0.518	1.15 (0.77–1.73)	0.492	1.12 (0.74–1.69)	0.588	1.15 (0.76–1.74)	0.515
Western	1.15 (0.78–1.70)	0.487	1.17 (0.79–1.72)	0.439	1.15 (0.77–1.70)	0.495	1.15 (0.78–1.71)	0.474	1.15 (0.78–1.70)	0.481	1.13 (0.77–1.68)	0.530	1.16 (0.78–1.72)	0.458
Eastern European	2.05 (1.41–2.98)	0.000	2.03 (1.39–2.97)	0.000	2.08 (1.43–3.03)	0.000	2.07 (1.42–3.01)	0.000	2.11 (1.45–3.08)	0.000	2.07 (1.42–3.02)	0.000	2.11 (1.44–3.11)	0.000
Latin American	1.85 (1.25–2.72)	0.002	1.81 (1.22–2.69)	0.003	1.83 (1.25–2.69)	0.002	1.81 (1.23–2.67)	0.003	1.95 (1.32–2.87)	0.001	1.82 (1.24–2.68)	0.002	1.84 (1.23–2.74)	0.003
Other Non-Western	1.50 (1.07–2.11)	0.018	1.54 (1.10–2.16)	0.013	1.47 (1.05–2.06)	0.026	1.49 (1.06–2.10)	0.020	1.59 (1.13–2.24)	0.008	1.50 (1.07–2.11)	0.018	1.56 (1.11–2.20)	0.011
**High Psychosoc. Demands**			2.00 (1.55–2.57)	0.000									1.82 (1.41–2.35)	0.000
**Low Decision Latitude**					1.40 (1.10–1.79)	0.007							1.18 (0.91–1.52)	0.205
**Low Social Support**							1.28 (0.98–1.66)	0.069					1.25 (0.96–1.64)	0.095
**Ergonomic Stress**									1.70 (1.32–2.21)	0.000			1.45 (1.08–1.94)	0.014
**Physical Demands**											1.57 (1.20–2.06)	0.001	1.21 (0.88–1.65)	0.238

* model adjusted for age, sex, occupational class, civil status, weekly working hours and migration reason.

The OR of mental distress are displayed in Table 3 by region of origin. Eastern European, Latin American and Other Non-Western workers had increased OR for mental distress when compared to Native Swedish workers after adjustment for age, sex, occupational class, civil status, weekly working hours and migration reason (Model 1). Associations remained significant in all subsequent models adjusted for working conditions (Models 2–7). All working conditions were found to be significantly associated with mental distress in all independent models apart from Model 4, in which only a trend towards significance was observed for exposure to low social support (*p* = 0.069). In the fully adjusted model, low decision latitude, low social support and physical demands were non-significant.

Table 4 shows explained fractions (XF) calculations which estimated the influence of working conditions on relationships between foreign background status and health. Overall, adverse working conditions did not appear to contribute to the risk for poor self-rated health among Eastern European and Other Non-Western workers. Among Latin American workers, in the fully adjusted model a modest 11% of the excess risk for poor self-rated health was attributable to adverse working conditions. Explained fractions calculations also suggested that exposure to adverse working conditions did not appear to impact the risk for mental distress among any of the foreign background groups.

**Table 4:** Explained fractions (XF) calculations of the influence of adverse working conditions on odds ratios of poor self-rated health and mental distress among foreign background workers in Sweden *.

	Model 2 *XF high psychosocial demands*	Model 3 *XF low decision latitude*	Model 4 *XF low social support*	Model 5 *XF ergonomic stress*	Model 6 *XF high physical demands*	Model 7 *XF all working conditions*
**Poor Self-Rated Health**						
Eastern European	0.02	N/A	N/A	N/A	N/A	N/A
Latin American	0.04	0.03	0.06	N/A	0.06	0.11
Other Non-Western	N/A	0.05	0.02	N/A	0.00	0.00
**Mental Distress**						
Eastern European	0.02	N/A	N/A	N/A	N/A	N/A
Latin American	0.04	0.02	0.04	N/A	0.03	0.01
Other-Non Western	N/A	0.07	0.02	N/A	0.00	N/A

* only groups with significantly increased OR for poor self-rated health and mental distress are shown; N/A denotes instances in which OR did not attenuate with the addition of working conditions variables into the model.

## 4. Discussion

The present study utilized cross-sectional survey data describing the living and working conditions of native and foreign background persons in Sweden to examine health outcomes and working conditions among these groups and investigate possible associations between exposure to adverse working conditions and risk for poor self-rated health and mental distress. Findings suggested that: (1) exposure to adverse working conditions varied by region of origin; (2) health inequalities were present between Native Swedish and some groups of foreign born workers; and (3) exposure to adverse psychosocial and physical working conditions had only a minimal influence on the increased risk for poor health found among some foreign born employees.

Consistent with previous research [14,30,42] several groups of foreign background workers were found to have greater exposure to adverse working conditions. Overall, Western and Eastern European workers appeared to have working conditions that were the most similar to Native Swedish workers. Other Non-Western, Foreign Parentage Swedish and Latin American workers experienced the highest proportions of physical demands and Other Non-Western workers also had the highest proportion of low decision latitude. However, Other Non-Western workers had the lowest exposure to high psychosocial demands. It may be the case that workers in this group are disproportionately segregated into lower status positions with more passive types of job duties and responsibilities which entail fewer psychosocial demands, as previous research has shown that persons coming from these countries are more likely to be confined to lower status jobs [43,44]. Individuals from these countries may also be more likely to migrate as refugees or asylum seekers, and as such may be forced into lower status jobs due to lasting psychological sequelae as a result of their trauma.

Our findings also demonstrate the persistence of health inequalities across two unique indicators of health and well-being among some foreign born workers in Sweden. Workers from Western countries and Foreign Parentage Swedish workers did not significantly differ from Native Swedish workers in their risk for both poor self-rated health and mental distress. Eastern European, Latin American and Other Non-Western workers had an increased risk of poor self-rated health compared to Native Swedish workers. This result is consistent with prior research in Sweden which has shown poorer self-rated health among some foreign born populations [30,45,46] and has important implications for health as well as labor market participation among some migrant groups, as good health is often necessary to obtain or retain employment. Eastern European, Latin American and Other Non-Western workers also had an increased risk of mental distress. This finding is in accordance with prior research which has also shown mental health disparities among some migrant background groups in Sweden, including persons from former Soviet countries [47], Latin American countries [48] and Other Non-Western countries [49,50] when compared to native born Swedish persons. As good mental health is widely recognized as a core component of overall health and well-being [51] and is vital in the successful management of working life stressors and demands [52], reducing mental health inequalities among the foreign born population could not only improve health outcomes but also labor market participation.

These findings also have potential implications for Swedish integration policies, under which labor market participation is given precedence as a key component of successful integration processes [53]. The foreign born participants in the present study are perhaps more integrated than those with weaker or more insecure labor market positions; only 8% worked less than 30 hours per week, and nearly 65% worked 40 hours or more. This strong labor market position further suggests that these individuals are perhaps also healthier than unemployed and underemployed foreign born persons in Sweden, as prior research has shown that an unstable or weak labor market connection can increase the risk of illness [54]. However, our results suggest the persistence of health inequalities even among foreign born workers with a secure labor market position. As such, integration policies which emphasize labor market attachment may not be sufficient to reduce health inequalities among foreign born populations and may need to pay increased attention to addressing structural and upstream factors that can impact both health and labor market attachment, such as social and economic policies, neighborhood factors, living conditions, discrimination and social capital [4].

Contrary to expectations, exposure to adverse working conditions did not appear to explain much of the increased risk for poor self-rated health and mental distress found among some foreign born workers in this study. Among Eastern European and Other Non-Western workers virtually none of the excess risk of poor health could be attributed to exposure to poor working conditions. Among Latin American workers only a minimal amount of excess risk for poor health could be attributed to exposure to adverse working conditions. Nonetheless, although exposure to poor working conditions did not appear explain health inequalities in this study it remains vital to improve working life conditions among foreign background workers as such conditions may influence other aspects of health and well-being than those assessed in the present study. In addition, it may be the case that there is a time lag between exposure to adverse working conditions and poor health outcomes which may not be apparent now but could become evident later in life.

As stated previously the foreign born individuals in this study are highly integrated, in that they had been living in Sweden for 22.5 years on average (SD = 11.1, range 5–63) at the time of survey administration and over 95% reported proficiency or fluency in Swedish. As such, the sample of foreign born workers included in the study may have a better health status or less exposure to poor working conditions than foreign born workers who have recently immigrated, have lower levels of Swedish language proficiency, or are underemployed (*i.e.*, those with temporary contracts or who work only part time) and may not be representative of all employed foreign born persons in Sweden. For example, more recently arrived individuals may have a weaker labor market position and thus be segregated into jobs which entail greater exposure to adverse working conditions [55,56]. More recent migrants could also have poorer health as a result of pre-, peri-, or post-migration stressors [57,58] and may therefore experience their work as more demanding or stressful, as prior studies have found associations between poor health and work absenteeism, performance and productivity [59,60]. Factors such as these may force less integrated migrants out of the labor market (temporarily or for longer periods of time) due to poor health status, poor working conditions, a weak labor market position or a combination of these factors. Alternately, it may be the case that foreign born workers are more vulnerable to other types of adverse working conditions or work related characteristics known to impact health which were not assessed in the present study, such as effort reward imbalance [61], job insecurity [62], or job satisfaction [63].

## 5. Limitations and Strengths

The findings of this study are tempered by several limitations and therefore must be interpreted with caution. First, cross sectional survey data was utilized, which does not allow for causality interpretations. For instance, it may be the case that persons with poorer health are segregated into positions which entail greater exposure to adverse working conditions, and as such we cannot assume that adverse working conditions lead to poor health. Similarly, we may also have problems with residual confounding as our data are not longitudinal. Second, the observed non-response rates suggest the presence of a possible response bias. Persons with poorer health may have been more likely to decline participation in the study, thus leading to an underestimation of ill-health prevalence. Third, the region of origin categories used in this study are somewhat broad and may mask differences between groups; however, the categorization method we utilized did enable us to compare groups by region of origin instead of merely analyzing the foreign born population as a whole. Fourth, cultural differences in illness symptomology experiences, conceptualizations of health and well-being, and stigma regarding mental health may have also influenced reporting. Differences in cultural attitudes which may shun types of reporting that could be viewed as complaining may have also impacted disclosure of poor working conditions or health ailments. However, as respondents in our sample were generally highly integrated the impact of cultural factors such as these was perhaps small. Fifth, this study did not examine the health status or working conditions of undocumented workers or foreign born persons who had lived in Sweden for less than five years. Such persons may differ from those in the present study in their health status, labor market attachment and working conditions. Finally, exposure to the most adverse working conditions may have been underestimated, as persons who have previously been injured on the job or experienced burnout due to poor working conditions may have been either forced out of the labor market because of their injury or condition (*i.e.*, the healthy worker effect [64,65]) or given less strenuous duties at the workplace until recovered.

The major strength of the present study is the utilization of a new and unique survey data set (LNU-UFB) which is the first of its kind to focus exclusively on examining the living and working conditions of the foreign born population in Sweden. Our research provides current and much needed evidence regarding the socio-demographic characteristics, health status and working conditions of employed foreign background persons in Sweden, which is necessary to inform public health and integration policies that promote health, equality, and well-being among this population.

## 6. Conclusions

This study represents an important first step in disentangling current relationships between migration, working life and health in Sweden and has demonstrated that health inequalities exist among foreign born workers. Although adverse working conditions only minimally influenced the excess risk for poor self-rated health and mental distress found among some groups of foreign born workers, the reduction of health inequalities and improvement of working conditions among foreign background populations should remain public health priorities. Foreign born persons are expected to comprise a larger proportion of the Swedish workforce in the coming years and a greater understanding of relationships between working life and health among foreign born persons and their children is needed. Further research should investigate whether or not exposure to adverse working conditions may have a greater health impact among less integrated foreign born workers or those who have weaker labor market attachments and also examine the role of other working life factors that could be detrimental to health among foreign background workers.

## References

[1] Mackenbach J.P. (2012). The persistence of health inequalities in modern welfare states: The explanation of a paradox. Soc. Sci. Med..

[2] Frieden T.R. (2011). Forward: CDC health disparities and inequalities report—United States, 2011. MMWR Surveill. Sum. (Washington, D.C.).

[3] Kautto M., Fritzell J., Hvinden B., Kvist J., Uusitalo H. (2001). Nordic Welfare States in the European Context.

[4] Rostila M., Toivanen S. (2012). Den Orättvisa Hälsan: Om Socioekonomiska Skillnader i Hälsa och Livslängd.

[5] Graham H. (2007). Unequal Lives: Health and Socioeconomic Inequalities.

[6] Commission on Social Determinants of Health, World Health Organization (2008). Fair Employment and Decent Work. Closing the Gap in a Generation: Health Equity through Action on the Social Determinants of Health.

[7] Marmot M., Wilkinson R.G. (2006). Social Determinants of Health.

[8] Cox T., Griffiths A., Rial-Gonzalz E. (2000). Research on Work-Related Stress.

[9] Robert Wood Johnson Foundation Issue Brief 4: Work Matters for Health. http://www.commissiononhealth.org/PDF/0e8ca13d-6fb8-451d-bac8-7d15343aacff/Issue%20Brief%204%20Dec%2008%20-%20Work%20and%20Health.pdf.

[10] Marmot M. (2004). The Status Syndrome.

[11] Kivimaki M., Virtanen M., Elovainio M., Kouvonen A., Vaananen A., Vahtera J. (2006). Work stress in the etiology of coronary heart disease—A meta-analysis. Scand. J. Work Environ. Health.

[12] Rostila M. (2008). The Swedish labour market in the 1990s: The very last of the healthy jobs?. Scand. J. Public Health.

[13] Rostila M. (2010). Birds of a feather flock together—And fall ill? Migrant homophily and health in Sweden. Soc. Health Illn..

[14] Nyampame I. (2008). Invandrade kvinnors hälsa i Sverige: Om genus och etnicitet. Soc. Tidskr..

[15] Daryani A., Berglund L., Andersson A., Kocturk T., Becker W., Vessby B. (2005). Risk factors for coronary heart disease among immigrant women from Iran and Turkey, compared to women of Swedish ethnicity. Ethn. Dis..

[16] Dotevall A., Rosengren A., Lappas G., Wilhelmsen L. (2000). Does immigration contribute to decreasing CHD incidence? Coronary risk factors among immigrants in Goteborg, Sweden. J. Intern. Med..

[17] Borne Y., Engstrom G., Essen B., Hedblad B. (2012). Immigrant status and increased risk of heart failure: The role of hypertension and life-style risk factors. BMC Cardiovasc. Disord..

[18] Albin B., Hjelm K., Ekberg J., Elmstahl S. (2005). Mortality among 723,948 foreign- and native-born Swedes 1970–1999. Eur. J. Public Health.

[19] Johansson B., Helgesson M., Lundberg I., Nordquist T., Leijon O., Lindberg P., Vingard E. (2012). Work and health among immigrants and native Swedes 1990–2008: A register-based study on hospitalization for common potentially work-related disorders, disability pension and mortality. BMC Public Health.

[20] Loeb S., Drevin L., Robinson D., Holmberg E., Carlsson S., Lambe M., Stattin P. (2013). Risk of localized and advanced prostate cancer among immigrants *versus* native-born Swedish men: A nation-wide population-based study. Cancer Causes Control.

[21] Statistics Sweden (2012). Statistical Yearbook of Sweden 2012: Population.

[22] Statistics Sweden (2011). The Future Population of Sweden, 2012–2060.

[23] Ingelby D. (2012). Ethnicity, migration and the 'social determinants of health' agenda. Psychosoc. Int..

[24] Bäckman O., Edling C., Marklund S. (2001). Work Environment and Work-related Health Problems in the 1990s. Worklife and Health in Sweden 2000.

[25] Hjerm M., Gustafsson R.A., Lundberg I. (2005). A Welfare State for Everyone? The Position of Immigrants In and Outside the Labour Market. Worklife and Health in Sweden 2004.

[26] Akhavan S., Bildt C., Wamala S. (2007). Work-related health factors for female immigrants in Sweden. Work.

[27] De los Reyes P. (2008). Etnisk Diskriminering i Arbetsliv: Kunskapläge och Kunskapsbehov.

[28] Rydgren J. (2004). Mechanisms of exclusion: Ethnic discrimination in the Swedish labour market. J. Ethn. Migr. Stud..

[29] Akhavan S., Bildt C.O., Franzen E.C., Wamala S. (2004). Health in relation to unemployment and sick leave among immigrants in Sweden from a gender perspective. J. Immgr. Health.

[30] Sundquist J., Ostergren P.O., Sundquist K., Johansson S.E. (2003). Psychosocial working conditions and self-reported long-term illness: A population-based study of Swedish-born and foreign-born employed persons. Ethn. Health.

[31] Johansson B., Vingård E. (2012). Migration, Arbetsmiljö, och Hälsa.

[32] Todorova I.L., Tucker K.L., Jimenez M.P., Lincoln A.K., Arevalo S., Falcon L.M. (2013). Determinants of self-rated health and the role of acculturation: Implications for health inequalities. Ethn. Health.

[33] Idler E.L., Benyamini Y. (1997). Self-rated health and mortality: A review of twenty-seven community studies. J. Health Soc. Behav..

[34] Fritzell J., Lundberg O. (2007). Health Inequalities and Welfare Resources: Continuity and Change in Sweden.

[35] Theorell T., Berkman L.F., Kawachi I. (2000). Working Conditions and Health. Social Epidemiology.

[36] Johnson J.V., Stewart W., Hall E.M., Fredlund P., Theorell T. (1996). Long-term psychosocial work environment and cardiovascular mortality among Swedish men. Amer. J Public Health.

[37] Karasek R., Theorell T. (1990). Healthy Work: Stress, Productivity, and the Reconstruction of Working Life.

[38] Ariëns G.A.M., van Mechelen W., Bongers P.M., Bouter L.M., van der Wal G. (2001). Psychosocial risk factors for neck pain: A systematic review. Am. J. Ind. Med..

[39] Karasek R. (1979). Job demands, job decisions latitude and mental strain: Implications for job redesign. Admini. Sci. Quart..

[40] Fredriksson K., Alfredsson L., Ahlberg G., Josephson M., Kilbom A., Wigaeus Hjelm E., Wiktorin C., Vingard E. (2002). Work environment and neck and shoulder pain: The influence of exposure time. Results from a population based case-control study. Occup. Environ. Med..

[41] Johnson N.J., Backlund E., Sorlie P.D., Loveless C.A. (2000). Marital status and mortality: The national longitudinal mortality study. Ann. Epidemiol..

[42] Schenker M.B. (2010). A global perspective of migration and occupational health. Amer. J. Ind. Med..

[43] Åslund O., Nordström Skans O. (2002). Will I See You at Work? Ethnic Workplace Segregation in Sweden, 1985–2002.

[44] Lundberg J. (2007). Working and Employment Conditions of Migrant Workers-Sweden.

[45] Leao T.S., Sundquist J., Johansson S.E., Sundquist K. (2009). The influence of age at migration and length of residence on self-rated health among Swedish immigrants: A cross-sectional study. Ethn. Health.

[46] Lindstrom M., Sundquist J., Ostergren P.O. (2001). Ethnic differences in self reported health in Malmö in southern Sweden. J. Epidemiol. Commun. H..

[47] Blomstedt Y., Johansson S.E., Sundquist J. (2007). Mental health of immigrants from the former Soviet Bloc: A future problem for primary health care in the enlarged European Union? A cross-sectional study. BMC Public Health.

[48] Sundquist J. (1994). Refugees, labour migrants and psychological distress. A population-based study of 338 Latin-American refugees, 161 South European and 396 Finnish labour migrants, and 996 Swedish age-, sex- and education-matched controls. Soc. Psych Psych Epid..

[49] Taloyan M., Sundquist J., Al-Windi A. (2008). The impact of ethnicity and self-reported health on psychological well-being: A comparative study of Kurdish-born and Swedish-born people. Nord. J. Psychiat..

[50] Steiner K.H., Johansson S.E., Sundquist J., Wandell P.E. (2007). Self-reported anxiety, sleeping problems and pain among Turkish-born immigrants in Sweden. Ethn. Health.

[51] Hermann H., Saxena S., Moodie R., World Health Organization (2005). Promoting Mental Health: Concepts, Emerging Evidence, Practice.

[52] Harnois G., Gabriel P., World Health Organization (2002). Mental Health and Work: Impact, Issues, and Good Practices.

[53] Regeringskansliet Swedish Integration Policy. http://www.government.se/content/1/c6/13/77/34/5b7683a6.pdf.

[54] Socialstyrelsen (2010). Social Report 2010: The National Report on Social Conditions in Sweden.

[55] Smith P.M., Mustard C.A. (2009). Comparing the risk of work-related injuries between immigrants to Canada and Canadian-born labour market participants. Occup. Environ. Med..

[56] Smith P.M., Chen C., Mustard C. (2009). Differential risk of employment in more physically demanding jobs among a recent cohort of immigrants to Canada. Inj. Prev..

[57] Newbold B. (2009). The short-term health of Canada's new immigrant arrivals: Evidence from LSIC. Ethn. Health.

[58] Yakushko O., Watson M., Thomspson S. (2008). Stress and coping in the lives of recent immigrants and refugees: Considerations for counseling. Int. J. Adv. Couns..

[59] Meerding W.J., IJzelenberg W., Koopmanschap M.A., Severens J.L., Burdorf A. (2005). Health problems lead to considerable productivity loss at work among workers with high physical load jobs. J. Clin. Epidemiol..

[60] Wynne-Jones G., Buck R., Varnava A., Phillips C., Main C.J. (2009). Impacts on work absence and performance: What really matters?. Occup. Med. (Lond.).

[61] Siegrist J. (1996). Adverse health effects of high-effort/low-reward conditions. J. Occup. Health Psychol..

[62] Laszlo K.D., Pikhart H., Kopp M.S., Bobak M., Pajak A., Malyutina S., Salavecz G., Marmot M. (2010). Job insecurity and health: A study of 16 European countries. Soc. Sci. Med..

[63] Faragher E.B., Cass M., Cooper C.L. (2005). The relationship between job satisfaction and health: A meta-analysis. Occup. Environ. Med..

[64] Li C.Y., Sung F.C. (2005). A review of the healthy worker effect in occupational epidemiology. Occup. Environ. Med..

[65] Östlin P. (1989). Occupational career and health: Methodological considerations on the healthy worker effect. Ph.D. Thesis.

